# Combretastatin-A4 disrupts neovascular development in non-neoplastic tissue

**DOI:** 10.1054/bjoc.2000.1653

**Published:** 2001-03

**Authors:** J Griggs, R Hesketh, G A Smith, K M Brindle, J C Metcalfe, G A Thomas, E D Williams

**Affiliations:** Department of Biochemistry, University of Cambridge, Tennis Court Road, Cambridge, CB2 1QW; Strangeways Research Laboratories, University of Cambridge, Wort's Causeway, Cambridge, CB1 8RN, UK

**Keywords:** angiogenesis, goitre, combretastatin, hyperplasia, tumour

## Abstract

Combretastatin-A4 phosphate (*cis* -CA-4) is a tubulin-binding agent currently undergoing clinical trials as an anti-tumour drug. We have investigated whether CA-4 functions as a tumour-specific anti-vascular agent using the hyperplastic thyroid as a novel in vivo model of neovascularization. CA-4 elicited pathological changes in normal tissue, manifested as the induction of multiple, discrete intravascular thrombi. These vascular-damaging effects indicate that CA-4P does not function as a tumour-specific agent but targets neovasculature irrespective of the primary angiogenic stimulus. © 2001 Cancer Research Campaign http://www.bjcancer.com


*British Journal of Cancer* (2001) **84**(6), 832–835
© 2001 Cancer Research Campaign
doi: 10.1054/ bjoc.2000.1653, available online at http://www.idealibrary.com on IDEAL®

http://www.bjcancer.com

### Combretastatin-A4 disrupts neovascular development in non-neoplastic tissue

**J Griggs[1], R Hesketh[1], GA Smith[1], KM Brindle[1], JC Metcalfe[1], GA Thomas[2] and ED Williams[2]**

[1]Department of Biochemistry, University of Cambridge, Tennis Court Road, Cambridge CB2 1QW; [2]Strangeways Research Laboratories, University of Cambridge, Wort's Causeway, Cambridge CB1 8RN, UK

**Summary** Combretastatin-A4 phosphate (*cis*-CA-4) is a tubulin-binding agent currently undergoing clinical trials as an anti-tumour drug. We have investigated whether CA-4 functions as a tumour-specific anti-vascular agent using the hyperplastic thyroid as a novel in vivo model of neovascularization. CA-4 elicited pathological changes in normal tissue, manifested as the induction of multiple, discrete intravascular thrombi. These vascular-damaging effects indicate that CA-4P does not function as a tumour-specific agent but targets neovasculature irrespective of the primary angiogenic stimulus. © 2001 Cancer Research Campaign http://www. bjcancer.com

**Keywords**: angiogenesis; goitre; combretastatin; hyperplasia; tumour

The prerequisite of vascularization to permit tumour development beyond a threshold size has focused attention on the therapeutic potential of anti-angiogenic and anti-vascular agents (Hanahan and Folkman, 1996; Hayes et al, 2000). *Cis*-CA-4, a tubulin-binding agent currently undergoing Phase I clinical trials, causes vascular disruption in some experimental primary and orthotopic tumours, and in vascularized metastatic tumour deposits (Dark et al, 1997; Beauregard et al, 1998; Grosios et al, 1999; reviewed in Griggs et al, 2001). Furthermore, *cis*-CA-4 inhibits metastasis of Lewis lung carcinoma. The *trans*-CA-4 isomer is without effect on either ectopic primary or metastatic Lewis lung carcinoma development (Griggs et al, manuscript submitted). *Cis*-CA-4 differentially affects quiescent and tumour vasculature, as indicated by the greater magnitude of transient changes induced in vascular pressure and resistance in tumours compared with those in normal tissues (Tozer et al, 1999). The mechanism of action of *cis*-CA-4 remains to be elucidated but it has been hypothesized that it manifests a selective toxicity for tumour endothelial cells (Dark et al, 1997), because their proliferative activity renders them more susceptible to the drug than the quiescent vasculature which predominates in the adult. The anti-vascular activities of *cis*-CA-4 have been reported to be specific for tumour vasculature. Here we examined this hypothesis by investigating whether proliferating endothelial cells in general are susceptible to CA-4P-mediated damage by studying its effects in rapidly proliferating, non-tumour tissue using chemical induction of goitre in mice.

A number of goitrogens can block thyroid hormone production leading to a rise in thyroid stimulating hormone (TSH) which promotes growth of thyroid follicular cells. This induces a co-ordinated programme of neovascularization in which endothelial cell proliferation is regulated by pro-angiogenic growth factors, for example vascular endothelial growth factor (VEGF), derived from follicular cells (Bidey et al, 1999). Following goitrogen treatment, thyroid growth is induced in rodents within 2 days and continues at a high rate for about 2 weeks. The weight of the gland approaches a plateau after about 4 weeks (Wynford-Thomas et al, 1982a; Peter et al, 1991). During the phase of active growth the mitotic activity in endothelial cells parallels that of epithelial cells (Wynford-Thomas et al, 1982b) and there is a very large increase in blood flow through the gland. We have utilized this system to compare the effects of *cis*-CA-4 and *trans*-CA-4 on normal and hyperplastic (goitrogen-treated) mouse thyroid.

*Received 14 September 2000*
*Revised 29 November 2000*
*Accepted 7 December 2000*

*Correspondence to:* TR Hesketh

### MATERIALS AND METHODS

### Induction of goitre

Male 8-week-old C57BL/6J mice (Charles River, UK) were housed according to institutional guidelines. 3 groups of 8 mice received drinking water containing 0.2% 3-amino-1,2,4-triazole (Fluka), 0.5% sodium perchlorate and 0.5% sucrose (BDH); 3 other groups of 7 mice received tap water.

### Combretastatin synthesis and administration

Both isomers of di-sodium phosphate CA-4 pro-drug were synthesized as previously described (Pettit et al., 1995, 1998; Bedford et al, 1996; Orsini et al, 1997; Pettit and Rhodes, 1998), dissolved in saline and filter sterilized. Goitrogen-treated and non-goitrogen-treated groups of mice were given either *cis*-CA-4 phosphate, *trans*-CA-4 phosphate or no further treatment. CA-4 was administered by intraperitoneal injection at a dose of 12.5 mg $kg^{-1}$ every 12 hours from the initiation of goitrogen treatment for 10 days, at which time all mice were killed by $CO_2$ asphyxiation. The treatment schedule used was selected based on previous studies (Griggs et al, manuscript submitted) in which a chronic treatment regime of 12.5 mg $kg^{-1}$ administered every 12 h by intraperitoneal injection is highly efficacious in the inhibition of metastatic development of Lewis lung carcinoma and manifests no detectable side effects or toxicity in C57B16/J mice.

### Immunohistochemistry

Thyroid glands attached to the trachea were removed, fixed in neutral buffered formalin (Sigma) for 24 h and embedded in paraffin. 4 μm thick sections were cut at 10 levels through each gland, taking 10 sections at each level. For each thyroid one section from each level was stained with von Willebrand factor (Dako) using an avidin-biotin-horse radish peroxidase technique with diaminobenzidine as the reporter molecule. All sections were screened microscopically at 100 × magnification by two observers without knowledge of the treatment groups to which they belonged.

### RESULTS

All goitrogen-treated groups showed diffuse thyroid enlargement with hypertrophic follicular cells, loss of colloid and reduction in size of follicular lumen, together with loss of the normal central and peripheral zonation (Figure 1B). All non-goitrogen-treated groups showed peripheral follicles with flattened epithelial cells, abundant colloid and more active central follicles (Figure 1A).

All animals in the *cis*-CA-4 groups given goitrogen showed multiple microthrombi either in small veins or capillaries within the thyroid (Figure 1E–H). No microthrombi were seen in extra-thyroid vessels or in a range of other normal tissues from the same animals (data not shown). In a few cases the microthrombi in capillaries had expanded to bulge into the lumen of an adjacent follicle, covered by a thin layer of stretched follicular cells. The incidence of microthrombi was quantified by counting the number of microthrombi detected in sections from 10 levels from each gland. The identification of microthrombi in stained sections was confirmed with von Willebrand factor antibody, which identified endothelial cells and also showed that the microthrombi contained the factor (Figure 1E, G, H). The abundant level of von Willebrand factor in the microthrombi may have originated from activated platelets in addition to damaged endothelial cells. In untreated goitre, endothelial cells were clearly identifiable by von Willebrand staining but were frequently undetectable at the periphery of microthrombi in *cis*-CA-4-treated goitres. Microthrombi were identified in the thyroids of all 8 mice treated with *cis*-CA-4, and were detected in 49 of 74 sections (66%). The mean number of microthrombi per goitre was 19.0 in *cis*-CA-4P-treated animals (Figure 2). None of the 8 animals treated with *trans*-CA-4 showed any microthrombi (Figure 1D) or any other change in any of the 74 sections, apart from those attributable to the goitrogen treatment. One of the 7 animals receiving goitrogen alone showed 3 microthrombi in 2 levels, no microthrombi were detected in the remaining 63 sections from this treatment group. We presume that this low incidence of spontaneously arising vascular disruption in untreated goitre arises from the hyperproliferative nature of the tissue. No other abnormalities were seen.

No microthrombi were seen in any of the mice not given goitrogen (Figure 2) whether treated with *cis*-CA-4 (Figure 1C) or *trans*-CA-4 (data not shown) or given no treatment (Figure 1A). All showed the expected normal gland morphology with no anomalous features.

### DISCUSSION

The data indicate that *cis*-CA-4 induces a high frequency of microthrombus formation in normal thyroid tissue that is undergoing a high rate of proliferation with associated neovascularization. Thyroid hyperplasia was induced by administration of a goitrogen: in the absence of this treatment *cis*-CA-4 had no detectable effects. The *trans* isomer of CA-4 was without effect on either normal thyroid tissue or in developing goitres, and neither isomer affected other normal tissues. The thyroid responses to CA-4 were consistent with the reported effects of the 2 isomers on primary and metastatic tumours in mice. *Cis*-CA-4 retards primary tumour growth by a process that involves severe vascular damage and it is also a potent inhibitor of metastatic development. The *trans*-CA-4 isomer, in contrast, does not inhibit either primary or secondary tumour development. *Cis*-CA-4 is a tubulin-binding agent but, unlike other agents with a similar action, for example, colchicine and vincristine, its action in vivo appears to be specifically against endothelial cells. In this study, the damage to the endothelial cells manifested itself as microthrombi in venules and capillaries.

Despite the frequency of microthrombus induction by *cis*-CA-4, there was no necrosis in any of the glands and no detectable change in the degree of hyperplasia in the follicular cells. The thyroid has a network of capillaries around the follicles and several arteries and veins which interconnect through this network. Thrombosis in all the major veins supplying the gland is therefore likely to be necessary before major changes are seen in the gland itself. In a rapidly growing tumour with new vessels growing into the expanding neoplasm, thrombosis in the newly formed vessels penetrating the tumour may well be more effective in causing necrosis or at least in slowing growth. We have no reason to suppose that vascular growth in the thyroid due to TSH stimulation differs in any fundamental aspect from angiogenesis in other tissues. During goitrogenesis vascular growth is coordinated, the thyroid endothelial cells responding to VEGF, as do endothelial cells of many other tissues, with VEGF being produced locally by follicular cells in response to TSH stimulation (Sato et al, 1995; Viglietto et al, 1997; Wang et al, 1998; Bidey et al, 1999). There is no evidence that the endothelial cells respond directly to TSH. Vascular growth in goitres is therefore considered to occur in response to stimulation from epithelial cells in a way that closely corresponds to angiogenesis in tumours induced by cancer cells. Goitrogenesis is a highly reproducible experimental system for inducing vascular growth and the stimulated thyroid gland has one of the highest rates of blood flow of any tissue. We have shown that the thyroid provides a reproducible in vivo model system for testing and quantifying the effects of putative anti-angiogenic or anti-vascular agents in non-tumour tissue.

The demonstration that *cis*-CA-4 induces widespread microthrombus formation in the rapidly growing vasculature of the stimulated thyroid indicates that its action is specific not for tumour vasculature but for growing endothelium. It follows that the drug may have considerable side effects if any non-neoplastic vascular proliferation is taking place, for example in inflammation. We note that the action of *cis*-CA-4 on hyperplastic, non-neoplastic vasculature suggests that this drug may be effective against angioproliferative diseases.

© 2001 Cancer Research Campaign

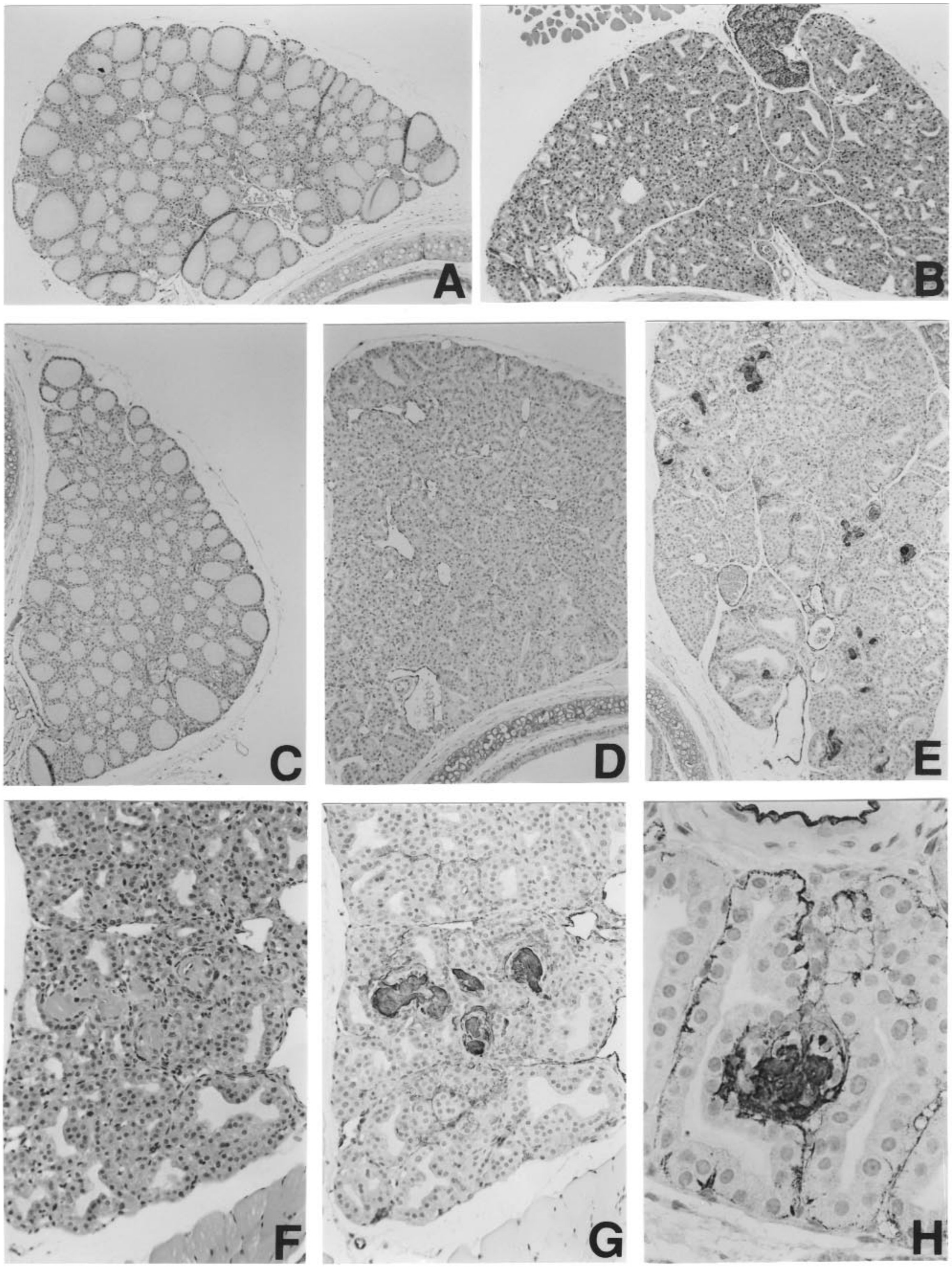

**Figure 1** Representative histological and immunohistochemical staining of mouse thyroid sections. (**A**) Section from an untreated mouse, showing normal thyroid architecture. A portion of the trachea is included (H&E × 60). (**B**) Section from a mouse treated with goitrogen only, showing diffuse thyroid hyperplasia. Part of the parathyroid is also present (H&E × 60). (**C**) Section from a mouse which was not given goitrogen to induce vascular and epithelial growth, but was treated with *cis* CA-4, showing no evidence of thrombi when stained with a rabbit polyclonal antibody (1:500) to von Willebrand factor (vWF) (× 60). (**D**) Section from a mouse treated with goitrogen and *trans*-CA-4, showing no thrombi (anti-vWF × 60). (**E**) Section from a mouse treated with goitrogen and *cis* CA-4, showing multiple thrombi which are positive for vWF (× 60). (**F** and **G**) Higher power photomicrographs from a mouse treated with goitrogen and *cis* CA-4 showing thrombi (H&E: **F**) which are positive for vWF (**G**) in the serial sections (both × 120). (**H**) A higher power photomicrograph from a mouse treated with goitrogen and *cis* CA-4, showing a von Willebrand factor-positive thrombus and perifollicular staining of the normal thyroid vasculature (× 360)

 © 2001 Cancer Research Campaign

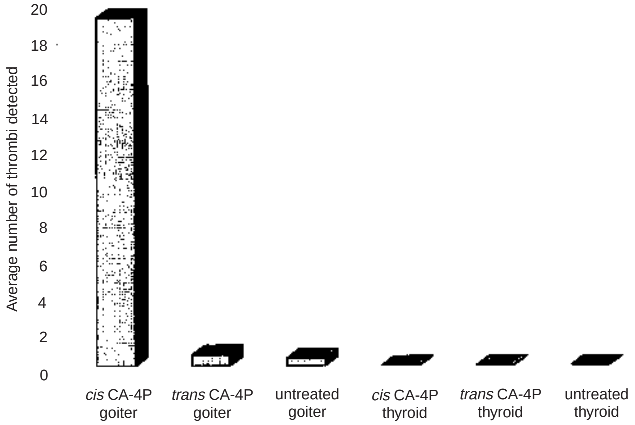

**Figure 2** Incidence of micro-thrombi in goitre and normal thyroid. The means of the aggregate number of thrombi from 10 levels of each gland ($n$ = 8) are shown. Mice were treated with *cis*-CA-4P, *trans*-CA-4P or untreated, with or without goitre induction

Preliminary evidence from Phase I clinical trials (reviewed in Griggs et al, 2001) suggests that CA-4 exerts an anti-vascular effect in some human primary tumours. Consistent with rapidly proliferating endothelium being the primary target of CA-4, we would speculate that there will be variability in the response of different tumour types to CA-4 according to the rates of vascular proliferation, a parameter which shows significant heterogeneity between tumours.

### ACKNOWLEDGEMENTS

We acknowledge Mrs Sally Davies for histological preparation and immunohistochemistry. J Griggs was supported in part by a BBSRC studentship.

### REFERENCES

Beauregard DA, Thelwall PE, Chaplin DJ, Hill SA, Adams GE and Brindle KM (1998) Magnetic resonance imaging and spectroscopy of combretastatin A(4) prodrug-induced disruption of tumour perfusion and energetic status. *Br J Cancer* **77**: 1761–1767

Bedford SB, Quarterman CP, Rathbone DL, Slack JA, Griffin RJ and Stevens MFG (1996) Synthesis of water-soluble prodrugs of the cytotoxic agent combretastatin A4. *Bio Med Chem Lett* **6**: 157–160

Bidey SP, Hill DJ and Eggo MC (1999) Growth factors and goitrogenesis. *J Endocrinol* **160**: 321–332

Dark GG, Hill SA, Prise VE, Tozer GM, Pettit GR and Chaplin DJ (1997) Combretastatin A-4, an agent that displays potent and selective toxicity toward tumour vasculature. *Cancer Res* **57**: 1829–1834

Griggs J, Metcalfe JC and Hesketh R (2001) Targeting tumour vasculature: the development of Combretastatin A4. *The Lancet Oncology* **2**: 82–87

Grosios K, Holwell SE, McGown AT, Pettit GR and Bibby MC (1999) In vivo and in vitro evaluation of combretastatin A-4 and its sodium phosphate prodrug. *Br J Cancer* **81**: 1318–1327

Hanahan D and Folkman J (1996) Patterns and emerging mechanisms of the angiogenic switch during tumorigenesis. *Cell* **86**: 353–364

Hayes AJ, Li LY and Lippman ME (2000) Anti-vascular therapy: a new approach to cancer treatment. *Western J Med* **172**: 39–42

Orsini F, Pelizzoni F, Bellini B and Miglierini G (1997) Synthesis of biologically active polyphenolic glycosides (combretastatin and resveratrol series). *Carbohydrate Res* **301**: 95–109

Peter H, Gerber H, Studer H, Groscurth P and Zakarija M (1991) Comparison of FRTL-5 cell growth in vitro with that of xenotransplanted cells and the thyroid of the recipient mouse. *Endocrinology* **128**: 211–219

Pettit GR and Rhodes MR (1998) Antineoplastic agents 389. New syntheses of the combretastatin A-4 prodrug. *Anti-Cancer Drug Design* **13**: 183–191

Pettit GR, Singh SB, Boyd MR, Hamel E, Pettit RK, Schmidt JM and Hogan F (1995) Antineoplastic agents 291. Isolation and synthesis of combretastatins a-4, a-5, and a-6. *J Med Chem* **38**: 1666–1672

Pettit GR, Rhodes MR, Herald DL, Chaplin DJ, Stratford MRL, Hamel E, Pettit RK, Chapuis JC and Oliva D (1998) Antineoplastic agents 393. Synthesis of the *trans*-isomer of combretastatin A-4 prodrug. *Anti-Cancer Drug Design* **13**: 981–993

Sato K, Yamazaki K, Shizume KYK, Obara T, Ohsumi K, Demura H, Yamaguchi S and Shibuya M (1995) Stimulation by thyroid-stimulating hormone and Graves' immunoglobulin G of vascular endothelial growth factor mRNA expression in human follicles in vitro and flt mRNA expression in the rat thyroid in vivo. *J Clin Investigation* **96**: 1295–1302

Tozer GM, Prise VE, Wilson J, Locke RJ, Vojnovic B, Stratford MRL, Dennis MF and Chaplin DJ (1999) Combretastatin A-4 phosphate as a tumour vascular-targeting agent: Early effects in tumours and normal tissues. *Cancer Res* **59**: 1626–1634

Viglietto G, Romano A, Manzo G, Chiappetta G, Paoletti I, Califano D, Galati M, Mauriello V, Bruni P, Laog C, Fusco A and Persico M (1997) Upregulation of the angiogenic factors PIGF, VEGF and their receptors (Flt-1, Flk-1/KDR) by TSH in cultured thyrocytes and in the thyroid gland of thiouracil-fed rats suggests a TSH-dependent paracrine mechanism for goitre hypervascularisation. *Oncogene* **15**: 2687–2698

Wang J, Milosveski V, Schramek C, Fong G, Becks G and Hill D (1998) Presence and possible role of vascular endothelial growth factor in thyroid cell growth and function. *J Endocrinol* **157**: 5–12

Wynford-Thomas D, Stringer B and Williams ED (1982a) Dissociation of growth and function in the rat thyroid during prolonged goitrogen administration. *Acta Endocrinol* **101**: 210–216

Wynford-Thomas D, Stringer B and Williams ED (1982b) Goitrogen induced thyroid growth in the rat: a quantitative morphometric study. *Acta Endocrinol* **94**: 131–140

© 2001 Cancer Research Campaign
